# Mechanical Characterisation and Analysis of a Passive Micro Heat Exchanger

**DOI:** 10.3390/mi11070668

**Published:** 2020-07-09

**Authors:** Francisco-Javier Granados-Ortiz, Joaquín Ortega-Casanova

**Affiliations:** Department of Mechanical, Thermal and Fluid Engineering, School of Industrial Engineering, University of Málaga, 29071 Málaga, Spain; jortega@uma.es

**Keywords:** micro heat exchanger, vortex shedding, thermal mixing, computational fluid dynamics (CFD), thermal engineering

## Abstract

Heat exchangers are widely used in many mechanical, electronic, and bioengineering applications at macro and microscale. Among these, the use of heat exchangers consisting of a single fluid passing through a set of geometries at different temperatures and two flows in T-shape channels have been extensively studied. However, the application of heat exchangers for thermal mixing over a geometry leading to vortex shedding has not been investigated. This numerical work aims to analyse and characterise a heat exchanger for microscale application, which consists of two laminar fluids at different temperature that impinge orthogonally onto a rectangular structure and generate vortex shedding mechanics that enhance thermal mixing. This work is novel in various aspects. This is the first work of its kind on heat transfer between two fluids (same fluid, different temperature) enhanced by vortex shedding mechanics. Additionally, this research fully characterise the underlying vortex mechanics by accounting all geometry and flow regime parameters (longitudinal aspect ratio, blockage ratio and Reynolds number), opposite to the existing works in the literature, which usually vary and analyse blockage ratio or longitudinal aspect ratio only. A relevant advantage of this heat exchanger is that represents a low-Reynolds passive device, not requiring additional energy nor moving elements to enhance thermal mixing. This allows its use especially at microscale, for instance in biomedical/biomechanical and microelectronic applications.

## 1. Introduction

Heat exchangers are present in many mechanical, biomechanical, and electronic engineering applications such as automobile refrigeration [1], air conditioning systems [2], powerplants [3], cooling of microelectronics [4], blood warming [5], or pressure ventilators [6,7]. Depending on the application, heat exchangers involving working fluids may aim at cooling/heating a fluid–fluid or fluid-solid system at different temperatures. The present investigation examines a fluid–fluid heat exchanger, which is influenced by an adiabatic fluid-structure interaction.

According to the transfer process, fluid–fluid heat exchangers can be classified into two groups. Indirect contact heat exchangers deal with heating/cooling by using a solid separation media between the two fluids. These are often called surface heat exchangers [8]. On the other hand, direct contact heat exchangers are systems where fluids have no physical separation, flowing within the same space [8]. An important point to consider in heat exchangers with thermal mixing of fluids is the ability to promote heat transfer at a low energy cost. The higher the mixing between the two flows at different temperature, the more efficient the thermal mixing is. Therefore, to find configurations that enhance mixing is the main objective for this type of heat exchangers. However, enhancing thermal mixing in fluids passing through channels is not cost-free. If moving elements are used (stirrers, heaving&pitching elements, etc.), one has to think of the energy needed for these elements to work. These devices that incorporate elements that consume electrical energy are called active devices, and these are a complex to include at microscale application. On the other hand, there is an alternative of using static mechanical devices, which do not require any additional energy consumption nor design of any moving element or structure at microscale. These are the namely passive devices. Examples of this type of device are the use of grooved channels or placing elements inside the channel, as in the present work. Although these new elements in the design do not need additional input energy, the pumping power $P_{p}$ is likely to be increased because of pressure loss is increased as a consequence of the drag force.

Several examples of the application of microscale heat exchangers and micromixers can be found in the literature. The main advantage of microdevices is that these offer a rapid, portable, simple, and low-cost tool [9]. For instance, Huang et al. [10] developed an experimental work for a microchannel heat transfer, where it was concluded that reentrant cavities are an ideal approach to improve the performance. In their work and review was outlined that to include elements to perturb the flow enhances heat transfer in microchannels. Pressure drop and heat transfer were analysed via simulation in Reference [11], where a single flow passed through a microchannel heat sink of a microelectronic device. In Reference [12] the thermal mixing of two fluids at different temperature was analysed theoretically, experimentally and numerically in a T-shaped microchannel at a very low Reynolds, and different volume flow-rate ratios were tested. Their results showed that the T-shape had two differentiated regions of heat exchange in terms of behaviour: the T-shape junction and the mixing channel flow. A very similar work in T-shape microchannels was developed in Reference [13] studying different temperatures and higher Reynolds numbers. A drawback in the use of T-shapes, F-shapes, etc. is that these increase dramatically the pumping power required to overcome such large pressure drop with respect to a straight microchannel, as shown by other authors [14,15]. In Reference [16] a gas-to-gas micro heat exchanger design with grooved channels was analysed experimentally. In their work, it was observed that when the volume flow rate is low, the impact of heat transfer by conduction was dominant within the wall and fins. During the literature review, the authors did not find any application of thermal mixing via micro heat exchangers enhanced by flow detachment from cylindrical/prism structures.

**Figure 1:**
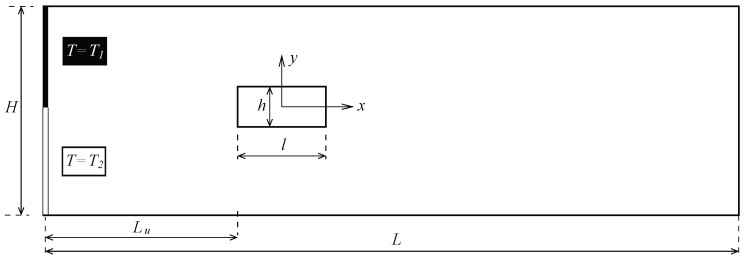
Sketch of the microchannel geometry with a rectangular structure positioning in the centreline.

The micro heat exchanger simulated (2D simulation), analysed and characterised for different configurations is depicted in Figure 1, where all geometric parameters are dimensional. This device consists of two flows at different temperatures (cold and hot, $T_{1}$ and $T_{2}$, respectively) passing through a microchannel with a rectangular cylinder structure (pillar) of width *h* and length *l*, located at the centreline. The microchannel has width *H* and total length *L*. Consequently, the cylinder-to-microchannel width ratio (blockage ratio) is $BR=\frac{h}{H}$; and the longitudinal cylinder aspect ratio, $AR=\frac{l}{h}$. The pillar structure is centered at the origin of the coordinates system, whose face nearest to the microchannel inlet is located at $L_{u}$ distance, and the face nearest to the microchannel outflow is positioned at a distance $L-(L_{u}+l)$ from it. As a consequence of this geometry, when the flow impinges on the structure for a certain geometrical configuration and flow regime, vortex shedding takes place, which leads to a thermal mixing between the hot and cold fluids.

Vortex shedding generated downstream cylinders has been extensively studied in the literature for more than a century. The first study on the problem dates back to 1907 to the pioneering work developed by Mallock [17]. Few years later, Benard continued on investigating the phenomenon [18,19]. Theodore von Karman probably did the most complete research on the topic back then, as shown in Reference [20]. Since these studies, many more researchers have investigated the mechanics behind this popular problem. Outlining the past-present history of the study of vortex shedding, one can find that in the last 5 years an impressive growing body of +16.400 research papers and patents have been developed on vortex shedding by cylinders. This highlights the importance the phenomenon still has in many engineering applications such as aircraft industry, heat exchangers, building engineering, civil engineering, environmental engineering or automobile industry, among many others.

Regarding the use of vortex shedding in heat transfer applications of laminar flows in channels, the interaction between a single flow and a cylinder at different temperatures is the most frequent application. This is opposite to chemical mixing applications, such as for instance Reference [21], where the molecular diffusion in a high Schmidt number flow was studied in the onset of the vortex shedding for a fixed value of BR and a fixed value of Re, and varying AR. Regarding the popular heat transfer interaction between cylinders in channels with laminar flows, in Reference [22] the convective heat transfer of an unsteady laminar flow (Re varied between 10 and 200) over a square cylinder in a channel (with a fixed BR=1/8=0.125 and AR=1) was analysed numerically. In this work, correlation models are found for the Nusselt-Reynolds number dependence, which is a regular practice in thermal engineering studies. It is relevant to outline that this type of model is often referred to as correlation in the engineering literature, but the correlation term will be used in this work joint with the term “model" as label to avoid any confusion with statistical correlation. However, there are no correlation models for other parameters such as pressure drop in any work in the literature of single-object vortex shedding in channels. Notwithstanding, one can find several works in the literature related to arrays of circular pins in microchannels, which are a good reference. For instance, Brunschwiler et al. [23] analysed heat-removal and pressure drop for arrays of in-line and staggered (circular) pins in chip stacks and plain parallel-wall microchannels, for which correlations were obtained. In their work, vortex shedding downstream was not addressed, and only weakly commented in terms of boundary-layer separation. Prasher et al. [24] analysed staggered arrays of circular pins with low pillar height aspect ratios in a microplate, and their experimental work was aimed at obtaining correlations for the Nusselt number and friction factor (which is related to the pressure drop) by including geometric and flow regime dependencies. Koşar et al. [25] also carried out an experimental investigation for low pillar height aspect ratio circular pin arrays. The experimental study was focused on determining correlation models for the pressure drop and friction factor. Focusing back again on single-object cases in channels, similarly to Reference [22], in Reference [26] the effect of channel confinement on the same vortex shedding heat transfer problem was analysed for Re=50,100 and 150 at different blockage ratio percentages and fixed AR=1, providing a comparison with the unconfined case. Similarly, in Reference [27] the effect of only varying the blockage ratio BR at different values for Re ranging between 62 and 300 was developed. As in most works in the literature, Turki et al. [27] studied variations on a AR=1 cylinder only, which was a limitation to generalise the multivariate nature of the problem to account also the AR effect. Moreover, as in other works found in the literature, this work stated different limits for the Reynolds number ($Re_{cr}$) above which the flow is unsteady. However, no correlation models as rule of thumb are given to know in advance whether a configuration may or may not lead to vortex shedding for the considered ranges. Surprisingly, only a few works were found in the literature related to the analysis of different AR values for laminar flows. Examples can be Reference [28], where the blockage ratio BR was fixed and AR was varied from 0.15 up to 4, for Re between 100 and 250 for a confined flow solved numerically; or Reference [29], where BR was fixed and different AR were ranged between 0.5 and 2, with Re between 50 and 200.

Unconfined flows have been also widely studied in the literature. For instance, Kelkar et al. [30] analysed a laminar flow which exchanged heat with a single heated square cylinder with Reynolds number Re ranged between 50 and 200. In such work was surprisingly stated that the heat transfer around the object is not pretty much enhanced with respect to steady cases when vortex shedding took place. This is opposite to our thermal mixing between two fluids. Different shapes have been also tested in the literature, not only the popular round or squared pillars. One can find, for instance, Reference [31], where a triangular cylinder shape was placed in a laminar regime channel to study the impact on heat transfer from the bottom side of the channel. Other similar applications of flow detachment in heat transfer can be found in the literature, such as Reference [32] where thermal-fluid-structure interaction was studied in a channel. In their research vortex shedding by means of flexible structure flags was used to promote thermal mixing between the cold flow and heated channel walls. Shi et al. [33] used a cylinder with a flexible plate to enhance heat transfer in a channel flow with heated walls by means of Vortex Induced Vibration (VIV). With this approach, a disruption of the thermal boundary layer by vortex interaction with the walls was aimed, and the use of vortex shedding mechanisms achieved an impressive heat transfer enhancement of up to a 90% with respect to a plain channel. In Reference [34] again a cold flow passed through a channel with flaps at the top and bottom of the heated channel walls. The flexible heated flaps were placed both symmetrically and asymmetrically, in order to study the kinematics of the flaps and to analyse the impact on the mixing promotion by vortex shedding. The vibrating flaps produced instabilities which strongly promoted mixing, and thence, thermal efficiency.

Summarising the literature review and research gaps found, some limitations have been identified. Apart from the absence of works on heat transfer between two microfluids at different temperature in the vortex shedding problem, it has been noticed an important gap in the characterisation of single-object vortex shedding in channels: there are no correlation models for relevant parameters such as the pressure related parameters (pressure drop, drag forces or the pumping power) nor the critical Reynolds (above which the flow is unsteady and von Karman streets do appear). Additionally, those works which intended to characterise and analyse the underlying mechanics of the problem did only focus efforts in indicating the limit values of one geometric factor at a time (and actually, only a couple of works investigated variations in AR), with poor generalisation. In the present investigation, a total amount of 80 simulations are developed (for Re∈[120,200], AR∈[0.125,1] and BR∈[0.2,0.5]). All the lacks described are addressed by providing correlation models between the pumping power, drag coefficient and critical Reynolds number $Re_{cr}$, by including all the geometric and regime parameters that govern the mechanics, as well as analysing the performance of the heat exchanger in thermal mixing.

The paper is divided into different sections as follows. Section 2 introduces the governing equations, defines the most relevant parameters and describes the numerical considerations for the computational simulation. In Section 3 the dependence of the critical Reynolds number with the geometric parameters, and the relations between other influential parameters of the micro heat exchanger set-up are analysed. Finally, in Section 4 the most relevant conclusions are given.

## 2. Computational Geometry and Numerical Approach

### 2.1. Governing Equations and Parameters

As shown in Figure 1, the inlet consists of two fluids (same fluid properties) at different temperature $T_{1}$ and $T_{2}$, whose velocity parabolic profile corresponds to a fully developed laminar flow. Their temperature $T_{i}$ is made dimensionless as $\theta_{i}$ by

(1) $$\theta_{i}\;=\;\frac{T_{i}-T_{1}}{T_{2}-T_{1}}\;=\;\frac{T_{i}-T_{1}}{\Delta T},$$

thus the inlet boundary condition is set as:

$$\theta \;=\;\theta_{1}\;=\;0\;\mathrm{at}\;x=-\left(L_{u}+l/2\right),\;0<y\leq H/2,$$

$$\theta \;=\;\theta_{2}\;=\;1\;\mathrm{at}\;x=-\left(L_{u}+l/2\right),\;-H/2\leq y\leq 0.$$

The thermal diffusivity of the fluids has been input to a very low value. Since the Prandtl number $Pr=\nu /\alpha_{t}$ is fixed at a high value of $10^{4}$, the thermal diffusivity of the flows is $\alpha_{t}\;=\;\nu \cdot 10^{-4}$, with ν the fluid kinematic viscosity. The reason of simulating the problem at such a low $\alpha_{t}$ value is that the heat exchange will be thus dominated by the vortex convective mechanics leading to fluid mixing. That is to say, by the designed geometry and flow regime. If $\alpha_{t}$ is chosen at a high value, then the heat exchanger performance would be less attributed to mixing mechanics and more attributed to fluid properties. The flow simulation is unsteady, 2D and the flow is incompressible. The pressure, *p*, and velocity, v=(u,v), fields are then governed by the Navier-Stokes equations in the dimensionless form:

(2) ∇·v=0,

(3) $$\frac{\partial v}{\partial t}+\left(v\cdot \nabla \right)v=-\nabla p+\frac{1}{Re}\nabla^{2}v,$$

whereas the mixing is governed by energy equation, written as

(4) $$\frac{\partial \theta }{\partial t}+\left(v\cdot \nabla \right)\theta =\frac{1}{Re\;Pr}\nabla^{2}\theta ,$$

where the viscous dissipation term has been neglected, due to the negligible value in comparison with the convection term. To transform all geometric and mechanical parameters into dimensionless quantities, the characteristic length, velocity, pressure and time used in the present work are *H*, *U*, $\rho U^{2}$ and H/U, respectively, with *H* the channel width, *U* the mean inlet velocity and ρ the density of the fluid. The Reynolds number defined in the equations above is Re=UH/ν. Thus, the geometric parameters are L=5H for the microchannel length, and $L_{u}=H$ for the pillar positioning respect to the inlet. The blockage ratio of the microchannel is defined as BR=h/H, and the aspect ratio is AR=l/h. When the regime is above a critical Reynolds value, $Re\geq Re_{cr}$, as a consequence of the oscillatory behaviour, the forces on the pillar structure are also oscillating. These forces are drag and lift, whose coefficients in dimensionless notation can be written as

(5) $$Cl=\frac{F_{y}}{\frac{1}{2}\rho U^{2}h},\;Cd=\frac{F_{x}}{\frac{1}{2}\rho U^{2}h},$$

where Cl and Cd are the lift and drag coefficients, respectively, and *h* is the pillar characteristic length, because in flows around objects this is the most appropriate length. The frequency of oscillation *f* is quantified by the dimensionless Strouhal number, St=fh/U. Since fluctuating quantities are complicated to analyse, it is useful to compute the time-averaged values, which vary according to its oscillation period $St^{-1}$. Thus, a time-averaged arbitrary quantity *M* can be computed as

(6) $$\langle M\rangle =\frac{1}{St_{M}^{-1}}\int_{t_{0}}^{t_{0}+St_{M}^{-1}}M\left(s\right)\;ds,$$

where $t_{0}$ stands for an initial time reference.

Forces on the rectangular pillar structure have a strong impact on the power requirements of the heat exchanger. The higher the drag force, the higher the pressure loss across the microchannel. This means that the pumping power required for the predicted performance would be also high. Such pressure loss can be modelled as a dimensionless pressure difference between the inlet and outlet of the microchannel. Thus, the dimensionless pumping power, denoted by $P_{p}$, can be calculated as

(7) $$P_{p}=2\Delta p\;q,$$

which is expressed in dimensionless form using $1/2\rho U^{3}H$ in the nondimensionalisation (the 1/2 is the reason of the 2 factor), and Δp and *q* stands for the dimensionless pressure drop and volume-flow rate, respectively. According to the characteristic quantities adopted in this work, the dimensionless volume flow-rate is q=1 (made dimensionless with UH), so the pumping power can be finally expressed as $P_{p}=2\Delta p$. In other works such as References [26,35] the pumping power is calculated as a function of the drag coefficient and Reynolds number, which is equivalent.

Since thermal mixing is the scope of the micro heat exchanger system, another important parameter is the assessment of the mixing quality. This feature must be evaluated at the outlet section, being defined as the thermal mixing efficiency η, in %, as the temperature deviation with respect to the average temperature (maximum standard deviation value possible) as done in previous literature:

(8) $$\eta =\left(1-\frac{\langle \sigma \rangle }{\frac{\theta_{2}-\theta_{1}}{2}}\right)\times 100,$$

where 〈σ〉 stands for the time average of the standard deviation of the dimensionless temperature θ at the microchannel exit. Since 0≤θ≤1, 〈σ〉=0 means that there is no deviation in temperature, thus the time-average temperature is unperturbed because the thermal mixing is perfect (η=100%). On the contrary, if the thermal mixing is as poor as there is no heat transfer between the microfluids at different temperature, the standard deviation with respect to the mean value is at its maximum value, 〈σ〉=0.5. Thus, in this case η=0%. Since very high values of the Prandtl number are used, the thermal mixing values are expected to be low. This means that if heat transfer is enhanced with the thermal mixing mechanism in this unfavourable case, for lower Prandtl fluids (for instance, air or water) the thermal mixing would be notably higher. Finally, both mechanical- and thermal-related properties, $P_{p}$ and η, can be combined into one parameter to define the thermal mixing energy cost (ϕ) as:

(9) $$\phi =\frac{P_{p}}{\eta },$$

which is the required pumping power to generate a 1% of thermal mixing efficiency. Mixing devices with high values of ϕ (high cost) are undesired, since this means high pumping power is required for a given mixing efficiency.

### 2.2. Numerical Aspects

The numerical investigation consists of a 2D computational mesh, whose governing equations are solved with the CFD software ANSYS-FLUENT 18.2. ANSYS-FLUENT allows the use of a pressure-based formulation for the incompressible flow, with a second-order spatial discretisation; and SIMPLE (Semi-Implicit Method for Pressure-Linked Equations) algorithm to deal with the pressure-velocity coupling. The boundary conditions imposed onto the numerical model in ANSYS-FLUENT (please see Reference [36] for further details on each boundary condition set-up) are:

- *Velocity-inlet*: For the laminar flow conditions, fully developed parabolic velocity profiles are imposed onto the simulation at the microchannel entrance, depending on each Reynolds number. For each fluid, a temperature is imposed (cold and hot), as aforementioned in Equation (1). Density and viscosity of both fluids is the same (related to each Reynolds number Re simulated), and a high Prandtl number of $Pr=10^{4}$ is also considered.
- *Pressure-outlet*: The pressure is imposed onto the outflow of the microchannel, imposing an atmospheric pressure. Transported quantities (let denote them by *M*) have gradients fixed to zero value: ∂M/∂x=0.
- *Wall*: A fixed zero heat flux (adiabatic surfaces) is imposed onto the upper and bottom walls of the microchannel, as well as onto the pillar structure: $\partial T(x_{s},y_{s};t)/\partial n\;=\;0$, where *n* stands for the coordinate normal to the considered wall surface and $\left(x_{s},y_{s}\right)$ is the exact position at the wall surface. The no-slip condition is also imposed in the velocity boundary condition on the wall: $v(x_{s},y_{s})\;=\;0$.

In addition to this set-up, the time discretisation step Δt has been selected such that the CFL number does not exceed the unity value: $u_{max}\Delta t/ds<1$. In the definition of the CFL number, since the mesh is uniform and has the same size in both *x* and *y* directions, thus Δx≡Δy≡ds. The $u_{max}$ term is the maximum velocity value in the computational domain.

In terms of validation of the 2D computational mesh, a mesh convergence analysis is a must. In this investigation, the Grid Convergence Index (GCI) developed by Roache [37] has been used. This method is a useful approach to measure the discretisation uncertainty based on the popular Richardson extrapolation. The objective of the GCI is to find an approximation for the exactness in the computation of a quantity of interest, obtained by different CFD grid refinements in a consistent and uniform basis. The method requires the computation in at least three different meshes, whose difference in results is contrasted one-by-one in increasing level of refinement. In GCI analysis, the mesh is halved in most works in the literature (which would mean a mesh refinement factor of 2), possibly in order to perform a reasonable systematic grid size reduction. Nevertheless, a refinement factor greater than 1.3 is recommended by Roache. An error estimation between grids is calculated by means of a generalised Richardson extrapolation, and a safety factor (recommended to be between 1.25 and 3 value in the literature) is applied to generate the grid uncertainty estimates. For further details, please see Reference [37].

**Figure 2:**
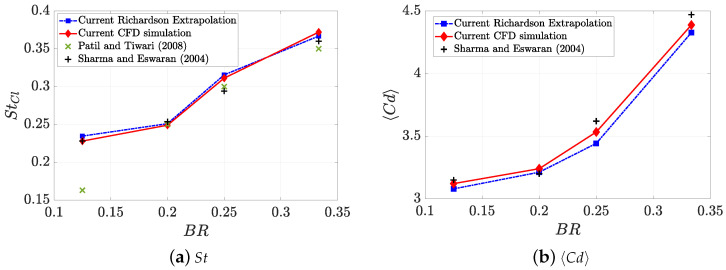
Validation of the CFD simulation with data from the literature: Patil and Tiwari (2008) [38] and Sharma and Eswaram (2004) [26]. The geometric configurations are confined flows around a square pillar (AR=1) and a fully developed flow at Re=100.

In this analysis, three different uniform structured meshes (with cell size $ds_{j}=0.0125$ H, 0.025 H and 0.05 H, namely by their indexes from fine to coarse as *j* = 1, 2, 3) have been tested for four different values of the blockage ratio (BR=0.125,0.2,0.25and0.33), a fixed Reynolds number (Re=100) and a fixed aspect ratio AR=1 (pillar of square section). The GCI results are presented in Table 1 as percentage for the Strouhal number of Cl ($St_{Cl}$, which is calculated from the frequency of the lift coefficient Cl time series) and 〈Cd〉. Additionally, the numerical results have been validated against experimental data, as shown in Figure 2, where also the Richardson extrapolated data (numerical value inferred if cell size tends to zero value) is shown.

**Figure 3:**
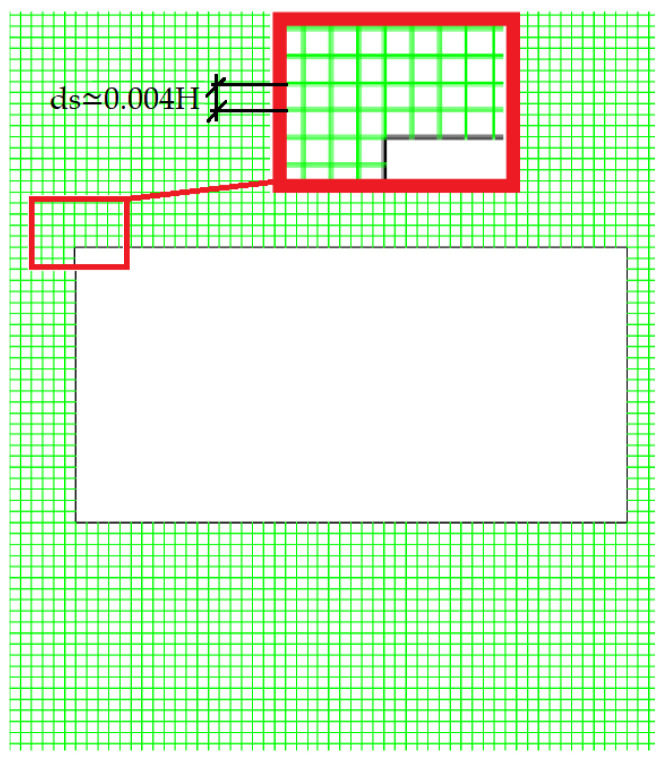
Detail of the uniform ds≃0.004 H mesh around a pillar with AR=2.

The parameter $GCI_{j+1,j}$ yields the discretisation uncertainty of each magnitude for the fine and medium grids with a safety factor of Fs=3, i.e., for j=1,2, since for the coarse mesh j=3 there is no previous computation to compare the convergence with. In Table 1 can be seen that the fine grid performed very well, with uncertainty ranging between 2.1% and 0.5% for 〈Cd〉; and between 1.9% and 0.5% for $St_{Cl}$. Thus, the fine mesh, with j=1, would be a very accurate option. However, the mesh must also be fine enough to solve the smallest thermal mixing length scales of θ. This is shown in the estimation of the Batchelor’s length scale Γ [39,40], which is the ratio between the length scale of the smallest velocity structures $\Gamma_{vel}$ and the square root of the Prandtl number: $\Gamma \;=\;\Gamma_{vel}/\sqrt{Pr}$. Since the size of the smallest structures in this laminar flow is of order *H*, then Γ∼0.01 H. This means that a finer mesh with a cell size smaller than $ds_{1}$ must be used. For that reason, the final mesh used to conduct the simulations was around 1/3 of the size of the fine mesh, i.e., ds≃0.004 H (see Figure 3).

## 3. Characterisation of the Micro Heat Exchanger

In many works in the literature (for instance Reference [27]), variations in the blockage ratio BR are studied and the cylinder is kept as square geometry. Other researchers just varied the flow regime by means of the Reynolds number Re to see above which values an unsteady flow appears. However, no correlation models as rule of thumb are given to know in advance whether a configuration may or may not lead to vortex shedding for considered ranges, nor the full geometric dependence on the performance of quantities of interest. An exception is the study using arrays of fin pins in Reference [41], where a threshold criteria to predict which cases would lead to vortex shedding was provided based on the geometric variables (transversal and longitudinal pitch ratio and height aspect ratio), although for a constant Reynolds number.

In several investigations on the same problem geometry studied in the present work (for instance Reference [22]) it is frequent to see correlation models, but only related to the heat transfer characteristics on the surface heat transfer exchange (frequently Nusselt number as function of Reynolds number). No correlation models dependent on all AR, BR and Re conditions are found for the quantities of interest in the literature. Moreover, Nusselt number is out of our scope, since the present investigation is only focused on thermal mixing. If other heat sources are introduced, may not be feasible to differentiate whether convective heat transfer intensification is being improved by thermal mixing or by heating/cooling the flow wake as a consequence of the interaction with a solid at different temperature or imposed heat flux. In the present investigation, the full geometric and regime dependence on the performance of the vortex shedding-based micro heat exchanger will be given in the following.

### 3.1. Geometry Leading to Vortex Shedding

The most interesting feature of this device is the beneficial use of the vortex shedding flow wake to enhance the thermal mixing between the two fluids confined in the microchannel. For this purpose, the geometry must be adequate, as well as the flow rate. Given a Re, AR and BR, vortex shedding from the pillar may or may not take place. If there is no oscillatory pattern, the flow is actually steady. Thence, for a given pair of values (AR,BR), there is a Reynolds number above which the flow is unsteady and oscillatory flow detachment is present. This is denoted as critical Reynolds number $Re_{cr}$: $Re_{cr}=Re_{cr}\left(AR,\;BR\right)$. Below this value, the flow is steady.

**Figure 4:**
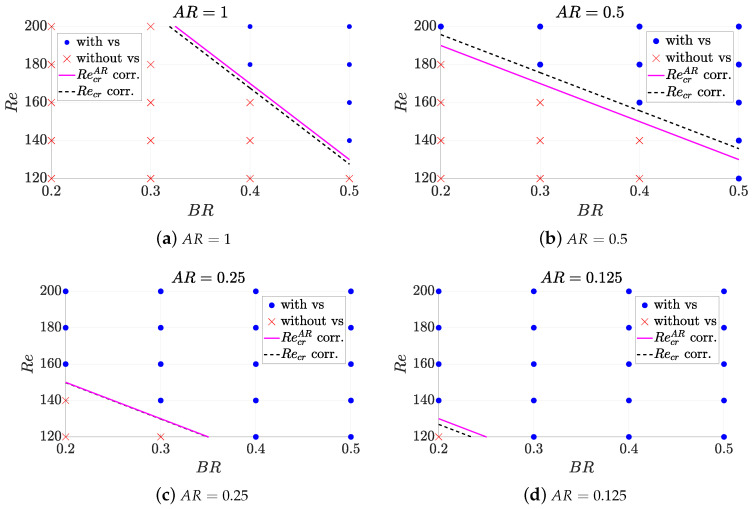
Impact of Reynolds number and geometry on the existence of vortex shedding (vs).

**Figure 5:**
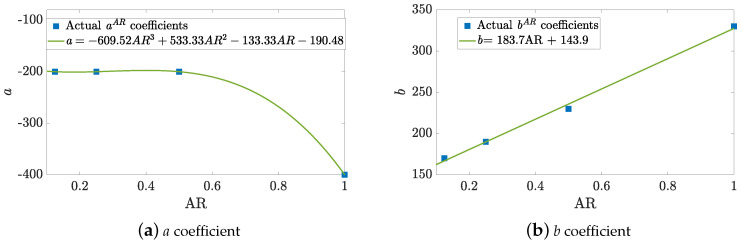
$Re_{cr}$ fitting coefficients.

This shows that it is crucial to determine the critical Reynolds according to the geometry in order characterise the micro heat exchanger. In Figure 4 it is illustrated the effect of Reynolds number and geometry on the generation of vortex shedding. It can be observed a limiting region of $Re_{cr}$ to classify those geometries leading to oscillatory pattern. A correlation model can be thus found for $Re_{cr}$ for characterisation. For each fixed value of AR, a different correlation $Re_{cr}^{AR}$ is found as illustrated by the magenta solid lines in Figure 4, whose equations correspond to:

(10) $$\begin{array}{c} Re_{cr}^{AR}=\;\left\{\begin{array}{c} -400\cdot BR+330\;\;\mathrm{if}\;\;AR=1,\\ -200\cdot BR+230\;\;\mathrm{if}\;\;AR=0.5,\\ -200\cdot BR+190\;\;\mathrm{if}\;\;AR=0.25,\\ -200\cdot BR+170\;\;\mathrm{if}\;\;AR=0.125, \end{array}\right. \end{array}$$

which are valid only for Re∈[120,200] and BR∈[0.2,0.5]. The correlation for $Re_{cr}^{AR}$ has a $Re_{cr}^{AR}\;=\;a^{AR}BR+b^{AR}$ linear form, being $a^{AR}$ and $b^{AR}$ coefficients which vary depending on AR values. With this approach, simple functions are used as correlation model; and only 1 case out of 80, the Re=120, AR=0.5 and BR=0.5 case, would be clustered incorrectly. These coefficients can be now approximated to obtain a $Re_{cr}$ non-linear correlation fully dependent on all the geometrical parameters within one single equation by finding the relationship of the coefficients above with respect to AR. Regression fits are obtained as shown in Figure 5 with a very accurate fit. Thus, finally a correlation for the $Re_{cr}$ can be found as

(11) $$Re_{cr}=aBR+b\;\left\{\begin{array}{c} \;a=-609.52AR^{3}+533.33AR^{2}-133.33AR-190.48,\\ \;b=183.7AR+143.9, \end{array}\right.$$

which is valid only for Re∈[120,200], AR∈[0.125,1] and BR∈[0.2,0.5]. The performance of this $Re_{cr}$ correlation is shown in Figure 4 by means of dashed black lines.

### 3.2. Mechanics of the Flow Around the Rectangular Structure

**Figure 6:**
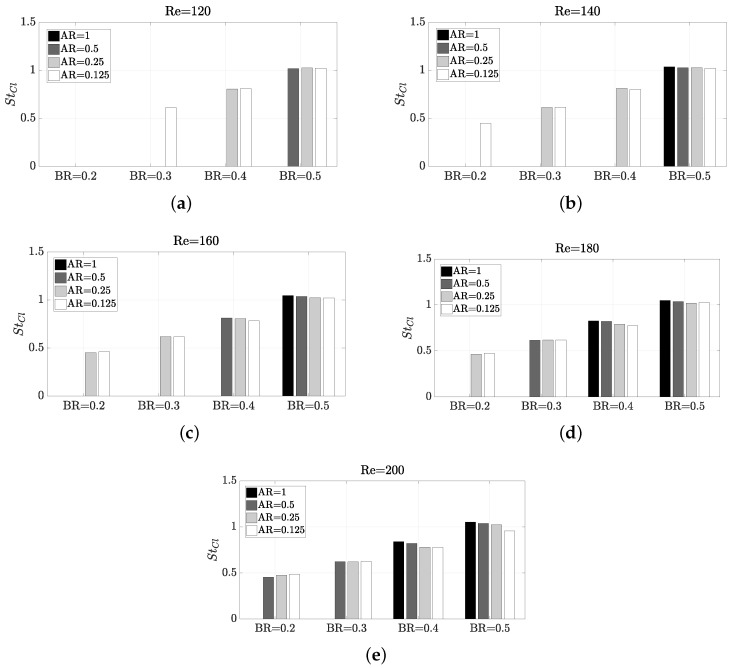
Impact of Reynolds number and geometry on Strouhal number in Cl.

When the flow impinges on the pillar, interesting structures are created. As a consequence, for Re values above the $Re_{cr}$, there is a signature oscillatory pattern for each configuration, which is shown on the Strouhal number. In Figure 6 is shown the behaviour of the Strouhal number for the lift coefficient $St_{Cl}$ with increasing Re, BR and AR. When the configuration does not lead to vortex shedding, the $St_{Cl}$ has zero value. An underlying linear behaviour is observed with respect to increasing BR, as already observed in Refenrence [27]. However, the effect of AR on the frequency is almost negligible. This is unexpected, because the aspect ratio obviously influences the existence of vortex shedding, but seems that this does not affect to the frequency value of the oscillation. This scenario also takes place with Re. If BR and AR are fixed and the Re is varied, it is observed that Re does not have any notable effect on the frequency either (although it affects the existence or not of the vortex detachment phenomenon, as seen in the $Re_{cr}$ characterisation study). In short, within the values considered in this work for the three parameters that define each possible configuration, if a low non-zero frequency of oscillation for the wake is desired, the best practice would be to keep a BR as low as possible. If a high frequency of oscillation is desired, a higher BR should be used.

**Figure 7:**
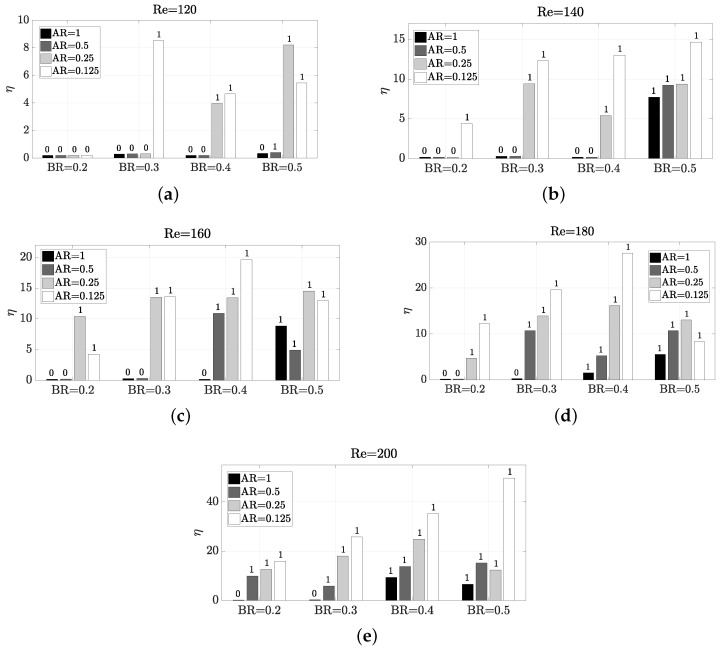
Impact of Reynolds number and geometry on thermal mixing efficiency η. The number on top of each bar indicates whether the configuration presents vortex shedding (1) or not (0).

**Figure 8:**
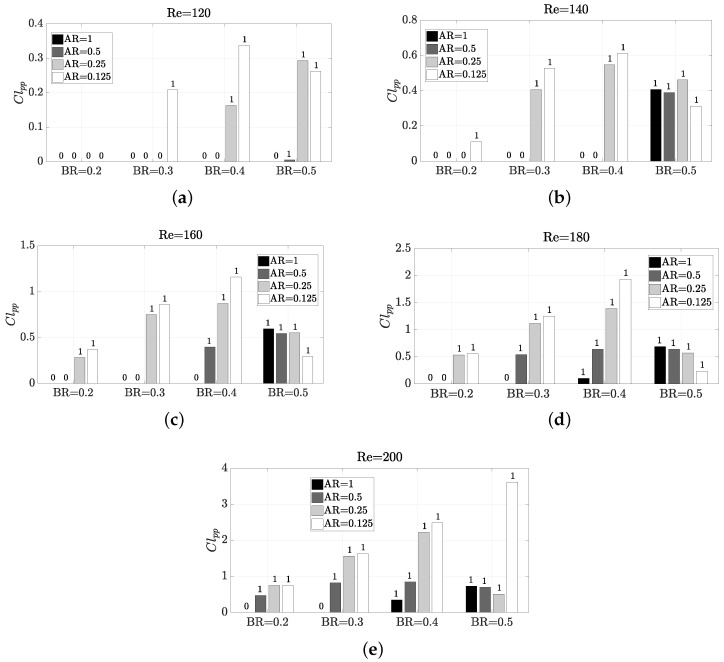
Impact of Reynolds number and geometry on the amplitude of the oscillation via $Cl_{pp}$. The number on top of each bar indicates whether the configuration presents vortex shedding (1) or not (0).

Since the thermal mixing efficiency η is enhanced by the vortex shedding, a similar behaviour to $St_{Cl}$ would be expected. However, by parameter exploration can be observed that the behaviour is very different. The parameter dependence pattern with η presents some sharp changes, as shown in Figure 7. The reason behind this nonexistent direct correlation between η and $St_{Cl}$ is that it is not only important the frequency of oscillation, but also the amplitude, since a high frequency may be achieved under low amplitude of oscillation. In this sense, a certain configuration $\left[Re,AR,BR\right]$ has different impact on frequency and amplitude, as shown in Figure 8, where the amplitude of the oscillation is observed on the peak-to-peak value of Cl ($Cl_{pp}$). As confirmed by a comparison of Figure 6 with Figure 7 and Figure 8, it is statistically correlated a high amplitude of oscillation with a high thermal mixing efficiency. Please note that the configurations with vortex shedding are indicated with ‘1’ on top of the bars, and with ‘0’ those which are steady. Thus, to find a configuration that enhances the amplitude of the frequency would lead to better thermal mixing results. The Pearson correlation coefficients *R* have been calculated to certify this, yielding a correlation between η and $Cl_{pp}$ of a 0.9421, whereas the correlation between η and $St_{Cl}$ was only a R=0.5728 (weakly correlated). We recall that the correlation coefficient ranges between −1 and 1, so a value close to R=1 means a high direct correlation. The correlation with $St_{Cl}$ is in practice misleading, because the very low oscillatory cases have zero or close-to-zero frequency values, but mixing is still taking place. The correlation without these zero-value-frequency cases in the data set (now only 53 cases) has a correlation coefficient of R=−0.0366 for $St_{Cl}$ (the $Cl_{pp}$ correlation coefficient is almost unperturbed when removing such data, with a R=0.9181 value).

**Figure 9:**
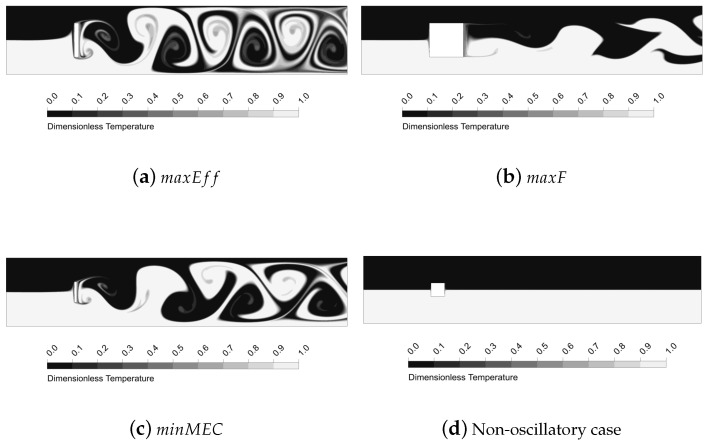
Thermal mixing performance for the micro heat exchangers maxEff, maxF, minMEC and a non-oscillatory case (AR=1, BR=0.2 and Re=200).

**Figure 10:**
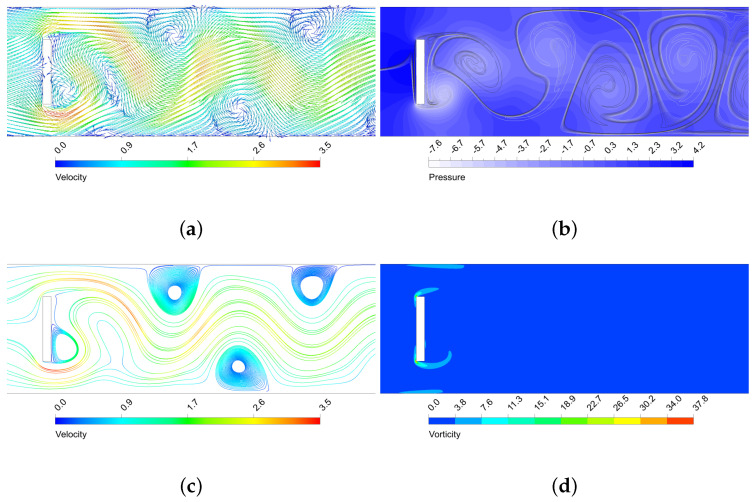
Performance of the maxEff micro heat exchanger near the rectangular structure. (**a**) Dimensionless velocity vector field; (**b**) Dimensionless static pressure (with mixing structures on top); (**c**) Streamlines (dimensionless velocity coloured scale); (**d**) Dimensionless vorticity.

**Figure 11:**
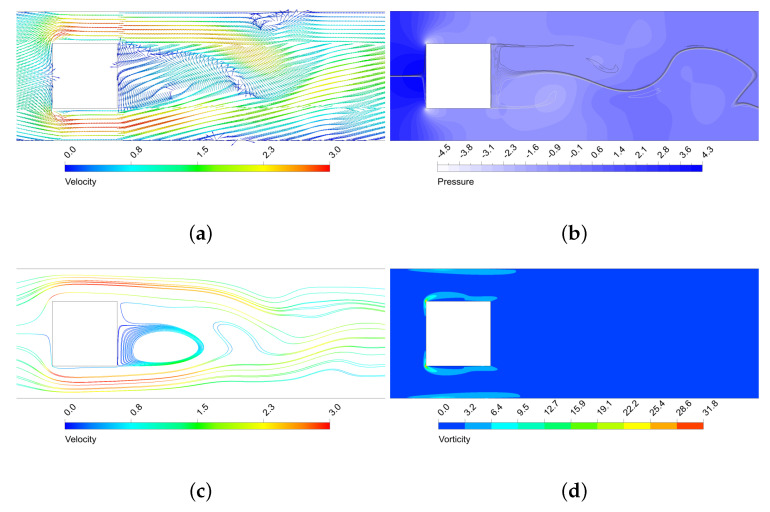
Performance of the maxF micro heat exchanger near the rectangular structure. (**a**) Dimensionless velocity vector field; (**b**) Dimensionless static pressure (with mixing structures on top) (**c**) Streamlines (dimensionless velocity coloured scale); (**d**) Dimensionless vorticity.

**Figure 12:**
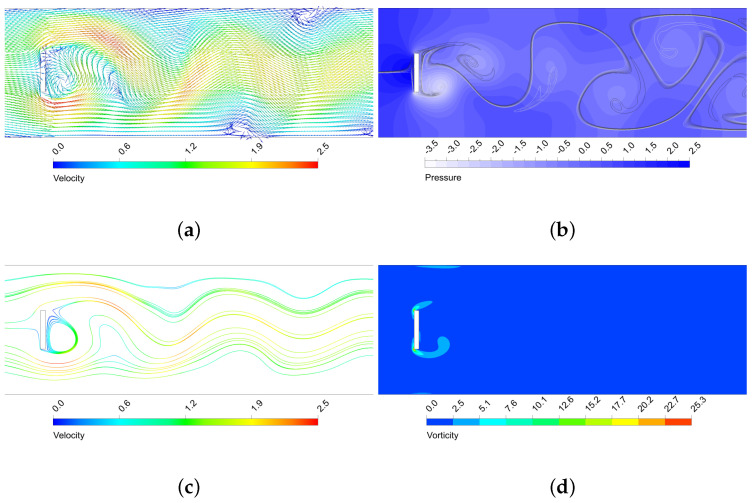
Performance of the minMEC micro heat exchanger near the rectangular structure. (**a**) Dimensionless velocity vector field; (**b**) Dimensionless static pressure (with mixing structures on top) (**c**) Streamlines (dimensionless velocity coloured scale); (**d**) Dimensionless vorticity.

The oscillatory motion of the fluid creates structures that enhance thermal mixing. As seen, the higher the amplitude of the vortex structures, the more enhanced the mixing is. In Figure 9, Figure 10, Figure 11 and Figure 12 is shown the behaviour of three configurations, denoted as maxEff, maxF and minMEC. maxEff is the configuration which led to the maximum thermal mixing efficiency (η=49.68%) among the 80 cases simulated, maxF is the configuration which led to the maximum frequency of oscillation ($St_{Cl}\;=\;1.0522$), and minMEC is the configuration that yielded the minimum mixing efficiency cost (ϕ=0.0540). Their characteristics are shown in Table 2.

By analysing the configurations in Figure 9, Figure 10, Figure 11 and Figure 12, it can be observed that the highest velocities are achieved by the maxEff configuration, which has a BR=0.5. maxF only differs with maxEff on the aspect ratio, but this shows a dramatically effect on the maximum velocity achieved (reduced by a 15%). As can be observed in Figure 10b, in maxEff the regions with higher velocity at the pillar object sides correspond to areas of very low pressure. This obvious fluid mechanics relation generates a suction effect on the high pressure upstream flow from the stagnation region, forming the beginning of the vortical structures that will potentially grow and travel downstream. This effect is strong for the vortex shedding thermal mixing: when the inlet flow impinges on the pillar structure, a portion of the hotter (colder) flow is able to pass through the upper (lower) side of the object (see the upstream curvature in the separation layer in Figure 9a). This occurs in an oscillatory manner, what enhances remarkably the thermal mixing and creates large vortical structures with low pressure cores. This situation does not take place for maxF, which has AR=1 and thence is not a sudden compression-expansion-like channel. In this case, the oscillatory motion has higher frequency, but very low amplitude of oscillation, since no large pressure gradients near the object take place to produce the aforementioned suction effect of hot (cold) fluids. Despite the vorticity generated by the corners is still remarkable, this is dissipated once the flow leaves the sides of the object, so the impact on the vortex shedding is low and nearly parallel to the pillar sides, as opposite to smaller AR cases.

By comparison of minMEC configuration with maxEff, where the only difference is in BR, one can see that the mixing is still good because large vortical structures are generated and propagated downstream. However, it can be observed a less influential role from the upper and lower walls of the microchannel: there is more separation between the thermal mixing flow and the channel walls (possibly because the upper and lower vortex shedding is taking place closer to the microchannel centreline, as BR is smaller). This generates less prominent recirculation zones near the microchannel wall in contrast to those that maxEff produced (see Figure 10c), which improve vorticity (and hence mixing), but require additional pumping power to drag the flow downstream. Also, last but not least, the drag force is dramatically reduced, since the face surface size of the pillar where the flow impinges is reduced by a 40%. In the next section will be shown the relation between the drag forces and pumping power for each configuration.

### 3.3. Analysis of Forces on the Rectangular Structure

**Figure 13:**
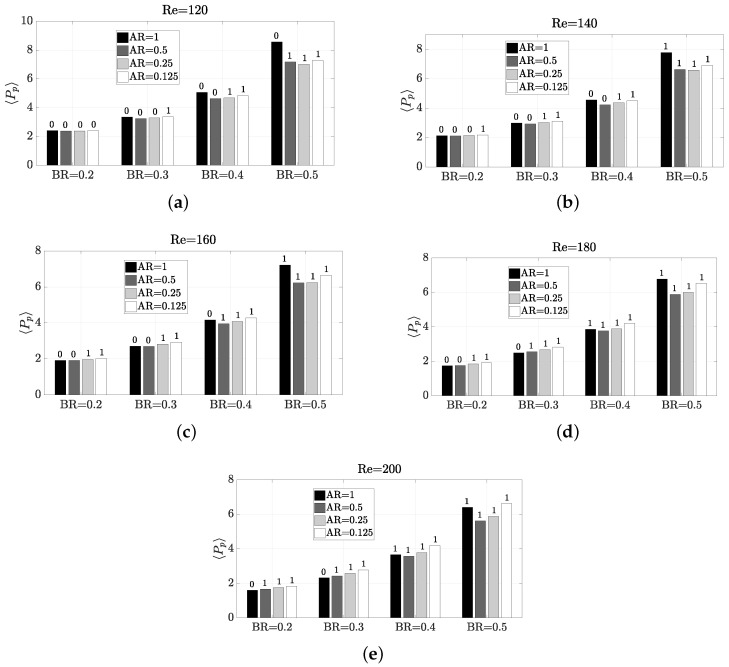
Impact of Reynolds number and geometry on the pumping power $\langle P_{p}\rangle$. The number on top of each bar indicates whether the configuration presents vortex shedding (1) or not (0).

The forces along the micro heat exchanger define the pumping power $P_{p}$ required to achieve the desired working conditions. The pumping power is plotted in Figure 13. As can be seen, the required pumping power is not very high. In fact, since this is strongly related to the pressure drop, a quick comparison with other pressure drop data from other microchannels reported in the literature is a valuable reference in Table 3. In such table, our dimensionless pressure loss ranges per unit length (made dimensionless with $\rho U^{2}/H$) are compared with those existing in a Y-shape microchannel [42] and a T-shape microchannel [43]. It is identified that among all the 80 cases simulated in the present work at various Reynolds and geometric configurations, all options had lower pressure loss than the Y and T-shape microchannels. For maxEff, maxF and minMEC, their dimensionless pressure loss per unit length is 0.661744, 0.6404 and 0.27772, which are actually very low values.

To predict and characterise the performance of a system is of high value in engineering practice. For this reason, the relationship between the pumping power, the Reynolds number and the geometry is analysed. The following expression allows to quantify such underlying relationship:

(12) $$\langle P_{p}\rangle \;=\;\left(\alpha_{1}BR^{\alpha_{2}}AR^{\alpha_{3}}+\alpha_{4}\right)\frac{1}{{\left(Re/100\right)}^{\alpha_{5}}},$$

where a vector for the fitting coefficients is defined as $\alpha \;=\;\left[\alpha_{1}\;\alpha_{2}\;\alpha_{3}\;\alpha_{4}\;\alpha_{5}\right]$. This expression is an empirical correlation model, which intends to predict the value of the pumping power based on relations between the geometric and flow regime parameters. Since the exact model is not known beforehand, some assumptions must be made. In the literature is very frequent to find terms using relations among power functions to account for non-linear interactions [25,41,44,45]. There are many works in the literature that explored empirical correlation models for the drag coefficient Cd of objects. In such investigations (see for instance Reference [45] for a review), the Reynolds number was always modelled as inversely proportional to the drag coefficient, as demonstrated more than a century ago by Stokes [46] and Oseen [47]. As $P_{p}$ and Cd are strongly related, the same modelling assumptions can be applied to $P_{p}$, leading to the correlation model suggested in Equation (12). Other researchers applied a similar logic to model the pressure drop and/or friction factor in the presence of arrays of pillars, as seen in References [23,24,25,41]. In Equation (12) the Reynolds number Re has been normalised with 100 to improve the numerical stability in the search of coefficients. It has been found by means of the Matlab *nlinfit* Least-Squares algorithm that the best coefficients are

(13) $$\alpha \;=\;\left[50.7966\;\;3.0216\;\;0.0312\;\;2.1216\;\;0.4520\right].$$

**Figure 14:**
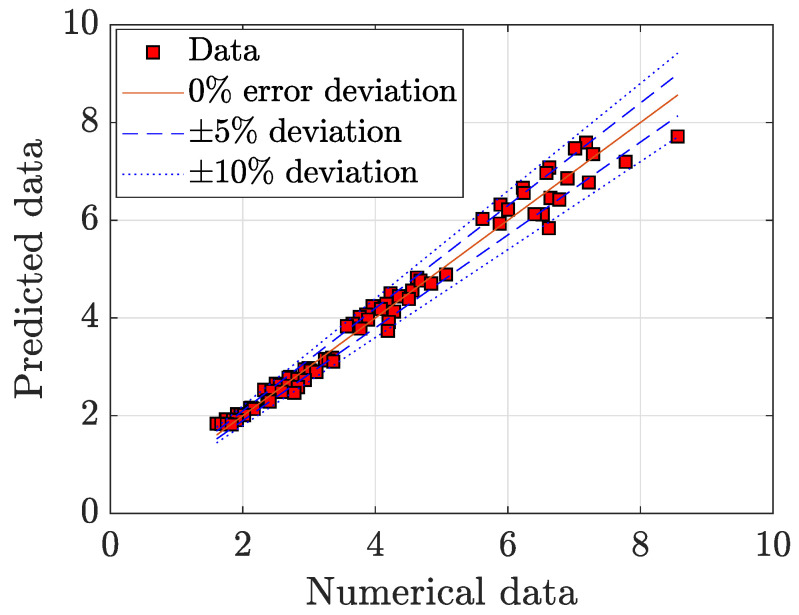
Fitting model for $P_{p}$.

This fit provided very accurate results, as shown in Figure 14. The correlation model yields a fitting error of 4.7202%, calculated for *N* samples as

(14) $$error\;\left[\%\right]\;=\;\frac{1}{N}\sum_{i=1}^{N}\left(\frac{|Predicted_{i}-Numerical_{i}|}{Numerical_{i}}\right)\cdot 100.$$

**Figure 15:**
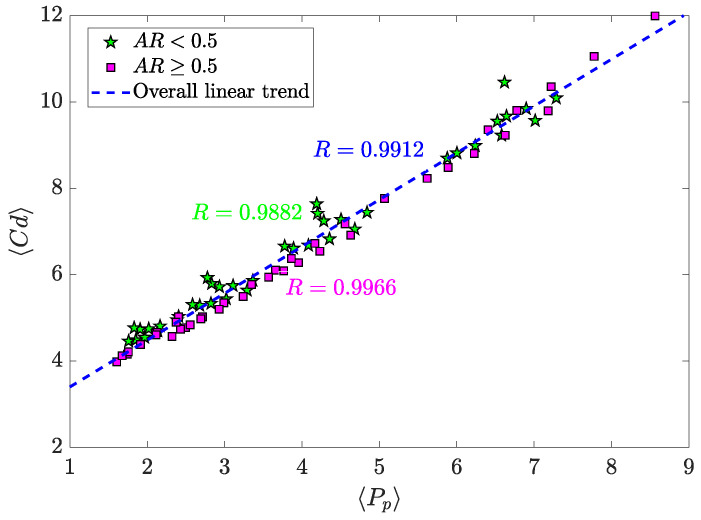
Pearson Correlation for $\langle P_{p}\rangle$ and 〈Cd〉. Please note that the correlation for each AR value has been obtained by including AR=0.5 in both greater and lower case scenarios, to have a greater number of data in each set.

Additionally, as mentioned above, $\langle P_{p}\rangle$ and 〈Cd〉 are related parameters. Their dependence is illustrated in Figure 15. It is shown that the correlation between these parameters is very high, which is evident since the higher the drag, the higher the pressure drop. However, it is less obvious the fact that these two parameters vary almost linearly, and when AR<0.5, data is more deviated from a linear trend. Nevertheless, the correlation coefficients are nearly invariant (R=0.9912 for any AR within the simulated range of values, R=0.9882 for AR<0.5, and R=0.9966 for AR≥0.5). This shows that the pumping power needed is almost entirely due to the drag force on the pillar structure, and the contribution to the pressure drop by the microchannel walls is very weak in comparison with such drag. Those cases of AR<0.5 that are more deviated from the overall linear trend are actually oscillatory cases of high amplitude. Thus, in these configurations the effect of the walls is contributing more to the pumping power requirement (the oscillatory flow “hits" the walls periodically), but still negligible.

**Figure 16:**
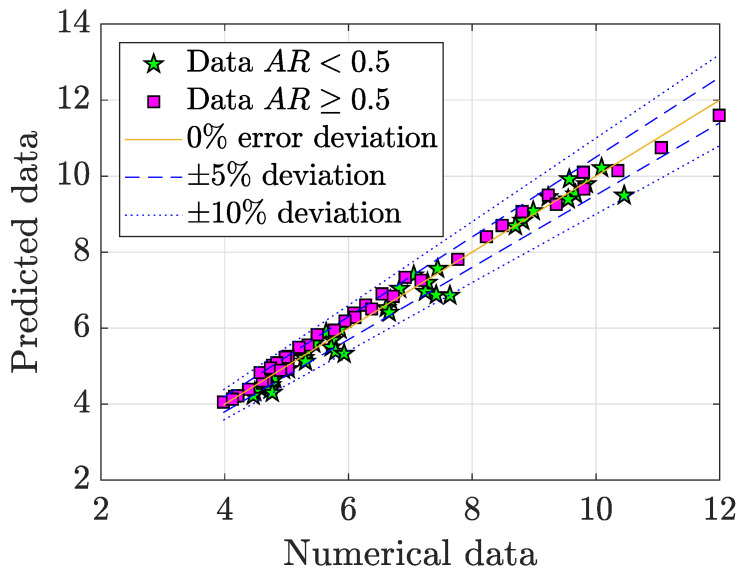
Model fit for 〈Cd〉 as a function of $\langle P_{p}\rangle )$.

Due to their linear underlying relationship, a linear correlation model can be developed to characterise their dependence. The model has the form:

(15) $$\langle Cd\rangle \;=\;\beta_{1}\langle P_{p}\rangle +\beta_{2},$$

and a vector with the fitting coefficients is defined as $\beta \;=\;\left[\beta_{1}\;\beta_{2}\right]$. The best fitting coefficients found are

(16) $$\beta \;=\;\left[1.0843\;\;2.3159\right],$$

with a fitting error of a 3.14%, as illustrated in Figure 16. In such figure can be observed that the data for AR<0.5 had worse fitting performance than AR≥0.5, because of the reasons mentioned above. Finally, since a model dependent on the regime and geometrical conditions was found in Equation (12) for $\langle P_{p}\rangle$, this can be substituted in Equation (16) to make 〈Cd〉 only dependent on Re, AR and BR.

### 3.4. Mixing Behaviour of the Micro Heat Exchanger

As aforesaid, thermal mixing in the micro heat exchanger is influenced by the type of flow (Pr), flow regime (Re), and geometry (AR, BR, *L*). The opposite effect to achieve a good mixing is the increase in pumping power requirement, which is influenced by the same parameters. Therefore, the mixing efficiency cost is a good ratio to evaluate the trade-off problem of achieving a good mixing at low pumping power cost. This ratio will be calculated per unit length *L*, since the length of the microchannel also plays a role in the thermal mixing:

(17) $$\phi_{L}\;=\;\frac{\phi }{L}.$$

**Figure 17:**
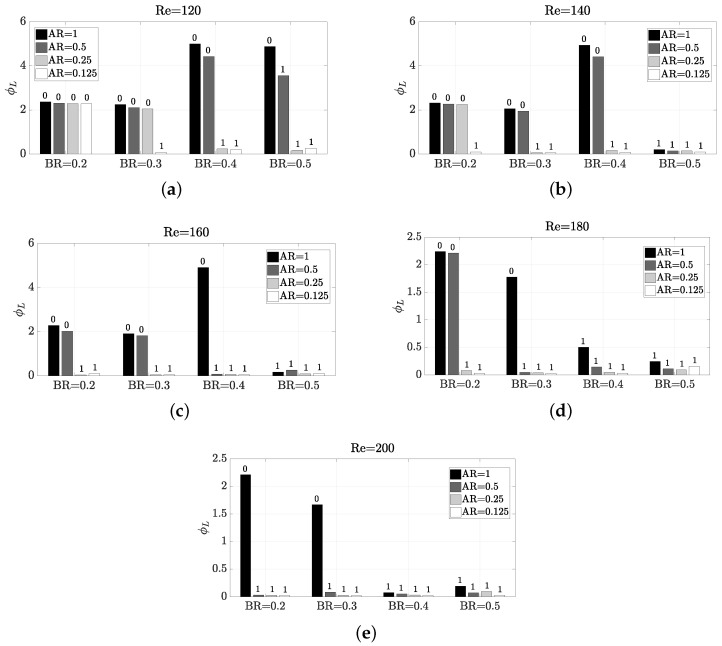
Impact of Reynolds number and geometry on $\phi_{L}$. The number on top of each bar indicates whether the configuration presents vortex shedding (1) or not (0).

Figure 17 details the operation of the micro heat exchanger in terms of $\phi_{L}$. It is obvious that the lower the $\phi_{L}$, the more cost efficient the heat exchanger is. Despite the underlying relations of $P_{p}$ with Re, AR and BR were clear and a model was built in Equation (12), the relationship of η with these parameters is not that clear. Thence, in $\phi_{L}$ it is not either possible to find any pattern to characterise the performance. From Figure 17 can be observed that all but one configurations that present vortex shedding have $\phi_{L}$ below 0.5. The only single case which experiences vortex shedding and $\phi_{L}$ is high, is Re=120, AR=0.5 and BR=0.5. For this configuration can be seen, despite that there is an oscillatory motion at high frequency (see Figure 6), and the amplitude is very low (see Figure 8). Also, the pumping power is large (see Figure 13). The observation that at 120≤Re≤160, BR=0.4 is the worst configuration possible is also interesting. This behaviour with BR=0.4 is apparently attributed to the fact that along the “sub-channels" created at the sides of the pillar structure, if BR is further increased, the flow is accelerated due to the section reduction, and the oscillatory motion starts. This lack of oscillation is worsened as AR is increased, since the flow expansion is less abrupt and the flow still have some time to adapt to the walls. Values of BR>0.5 are not considered in this study, since the increase in pumping power is significant. Therefore, a weak generalised aspect is that large values of AR need more cost for an efficient thermal mixing. This is especially notable for the higher Reynolds numbers considered, where the combination of low BR and high AR is very undesired.

## 4. Conclusions

This paper investigated the performance of a microscale heat exchanger, which consists of a rectangular pillar structure in a microchannel, where two fluids at different temperature are mixing. This represents a low-cost passive thermal mixing microdevice very appropriate for microscale applications, since no moving parts are required for heat transfer enhancement. Besides the heat transfer problem, the mechanics of the vortex shedding have been characterised. Opposite to the existing works in the literature of single-object confined vortex shedding, which do not consider more than two design parameters, a large number of different configurations varying simultaneously the longitudinal aspect ratio, blockage ratio and Reynolds number have been simulated. By means of empirical models and analysis of relevant contours and plots, the underlying relations between these parameters (including critical Reynolds number values) have been analysed. One of the most interesting features observed in the mechanism is that, for configurations with at least moderate oscillation, a pressure suction-like effect takes place periodically, allowing a portion of the hotter (colder) flow to pass through the upper (lower) side around the object and enhancing the thermal mixing.

For an efficient mixing configuration must be taken into account that large values of AR need more pumping power cost to achieve an efficient thermal mixing, as shown by the mixing efficiency cost per unit length. The impact of BR is less clear: to increase its value is usually beneficial for the thermal mixing efficiency, but the pumping power is increased notably. Thus, it is not possible to generalise the conclusions to a given specific rule of thumb parameter configuration. In this investigation, the best combinations for the considered parameter ranges seem to take place mostly for large Re, large BR and small AR. However, this generalisation is not always true, as observed for instance in the thermal mixing efficiency. For this parameter, at Re=200 the statement is true, but if Re=180, a BR=0.5 is less beneficial than a BR=0.4. This shows the complexity of the micro heat exchanger mechanics and the need for a careful design and testing by engineers.

## Figures and Tables

**Table 1:** Mesh convergence study (GCI).

			${\mathrm{St}}_{\mathrm{Cl}}$	〈Cd〉
Re	BR	Grid: *j*	${\mathrm{GCI}}_{j+1,j}$	${\mathrm{GCI}}_{j+1,j}$
100	0.125	1	1.0%	2.1%
		2	2.9%	4.6%
100	0.2	1	0.5%	0.6%
		2	2.6%	1.8%
100	0.25	1	1.9%	0.5%
		2	4.5%	4.1%
100	0.33	1	1.0%	0.6%
		2	4.1%	4.2%

**Table 2:** Efficient configurations for thermal mixing enhancement.

Configuration Label	AR	BR	Re	η	〈Δp〉	${\mathrm{St}}_{\mathrm{Cl}}$	ϕ
maxEff	0.125	0.5	200	49.6870%	3.30872	0.9549	0.0666
maxF	1	0.5	200	6.6167%	3.2020	1.0522	0.4839
minMEC	0.125	0.3	200	25.7284%	1.3886	0.6242	0.0540

**Table 3:** Comparison of pressure losses with different mixing microchannels.

	[42], Re=50	[43], Re=200	Current Research, Re=[120,200]
Δp/L [-]	0.93–3.59	1.23–23.52	0.16052–0.85624
